# Bacterial Antifouling Characteristics of Helicene—Graphene Films

**DOI:** 10.3390/nano11010089

**Published:** 2021-01-03

**Authors:** Shuhao Liu, Michael Bae, Li Hao, Jun Kyun Oh, Andrew R. White, Younjin Min, Luis Cisneros-Zevallos, Mustafa Akbulut

**Affiliations:** 1 Artie McFerrin Department of Chemical Engineering, Texas A&M University, College Station, TX 77843, USA; liushuhao1993@tamu.edu (S.L.); bsy7790@tamu.edu (M.B.); 2 School of Chemistry and Chemical Engineering, Zhongkai University of Agriculture and Engineering, Guangzhou 510225, China; haoli20@tju.edu.cn; 3 Department of Polymer Science and Engineering, Dankook University, 152 Jukjeon-ro, Suji-gu, Yongin-si 16890, Korea; junkyunoh@dankook.ac.kr; 4 Department of Chemical and Environmental Engineering, University of California, Riverside, CA 92521, USA; andrew.white@ucr.edu (A.R.W.); ymin@engr.ucr.edu (Y.M.); 5 Department of Horticultural Sciences, Texas A&M University, College Station, TX 77843, USA; lcisnero@tamu.edu

**Keywords:** bacterial antifouling, helicene, thin film, Langmuir-Schaefer, *S. aureus*

## Abstract

Herein, we describe interfacially-assembled [7]helicene films that were deposited on graphene monolayer using the Langmuir-Schaefer deposition by utilizing the interactions of nonplanar (helicene) and planar (graphene) π–π interactions as functional antifouling coatings. Bacterial adhesion of Staphylococcus aureus on helicene—graphene films was noticeably lower than that on bare graphene, up to 96.8% reductions in bacterial adhesion. The promising bacterial antifouling characteristics of helicene films was attributed to the unique molecular geometry of helicene, i.e., nano-helix, which can hinder the nanoscale bacterial docking processes on a surface. We envision that helicene—graphene films may eventually be used as protective coatings against bacterial antifouling on the electronic components of clinical and biomedical devices.

## 1. Introduction

Helicenes are an intriguing class of materials involving ortho-condensation of benzene or similar aromatic rings to form polycyclic aromatic nanoarchitectures with nonplanar, helical (screw-shaped) geometries [1]. In spite of the absence of asymmetric carbons or other chiral centers, helicenes still demonstrate chirality owing to their axial chirality [2]. The torsional strain of helical nanoarchitecture leads to different C–C bond lengths in the inner and outer helix: C–C bond length is about 0.143 nm for the inner ones while it is 0.135 nm for the outer ones [3]. These unusual properties have resulted in the development of their applications as asymmetric catalysts [4], magnetic spin filters [5], dyes [6], and DNA binders [7].

The presence of the nonplanar π–groups of helicene has been utilized for binding and self-assembly of helicenes on metals via metal–π interactions and on carbon nanomaterials via π–π interactions [8,9,10]. Feng et al. [11] have utilized hydrophobic interactions to deposit amide-containing helicenes on alkylated quartz via the Langmuir-Blodgett technique. Some other helicenes that are used for the production of Langmuir-Blodgett thin films include 2-amino[6]helicene derivatives [12], thiohelicene bisquinone [13], and dibenzo[6]helicene derivatives [14].

Bacterial contamination is a growing global problem adversely affecting the performance and function of many devices, coatings, and systems as well as causing bacterial infections [15,16,17,18]. In particular, *Staphylococcus aureus* (*S. aureus*) is a bacterial pathogen commonly responsible for various types of human infections such as skin, bone, bloodstream, lung and respiratory tract infections, and device-related infections [19,20,21,22,23]. *S. aureus* is the primary cause of surgical site infections, cutaneous abscesses, and purulent cellulitis [24]. *S. aureus* bacteremia, which has an incidence rate of 20 to 50 per 100,000 people per year, is a problematic infection with a high rate of morbidity and mortality (in the range of 10% to 30%) [25]. *S. aureus* is the most common cause of infective endocarditis with an incidence of the rate of 1.5 to 6 per 100,000 people per annum in Europe and the United States [26].

Owing to a large number of infections and associated economic losses stemming from bacterial adhesion and fouling, the development and discovery of novel bacterial antifouling surfaces is a topic receiving increasing attention from scientists and researchers [27,28,29,30,31,32,33,34,35,36,37]. While various applications of helicenes have been reported before, no prior work, to the best of our knowledge, has focused on the interactions of bacteria with helicene films. In this work, thin films of helicene on graphene with varying packing density were prepared using the Langmuir-Schaefer technique while monitoring their pressure-area isotherms. The morphology and wetting characteristics of these films were characterized by atomic force microscopy (AFM) and contact angle measurements. Bacterial adhesion on these films was directly measured using scanning electron microscopy (SEM) upon dip-inoculation. Most importantly, we herein demonstrate the proof-of-concept that [7]helicene coated graphene monolayer (H-GR) have promising bacterial antifouling properties with a reduction in the bacterial adhesion of *S. aureus* up to 96.8% compared to bare graphene surfaces.

## 2. Methods and Experiments

**Figure 1:**
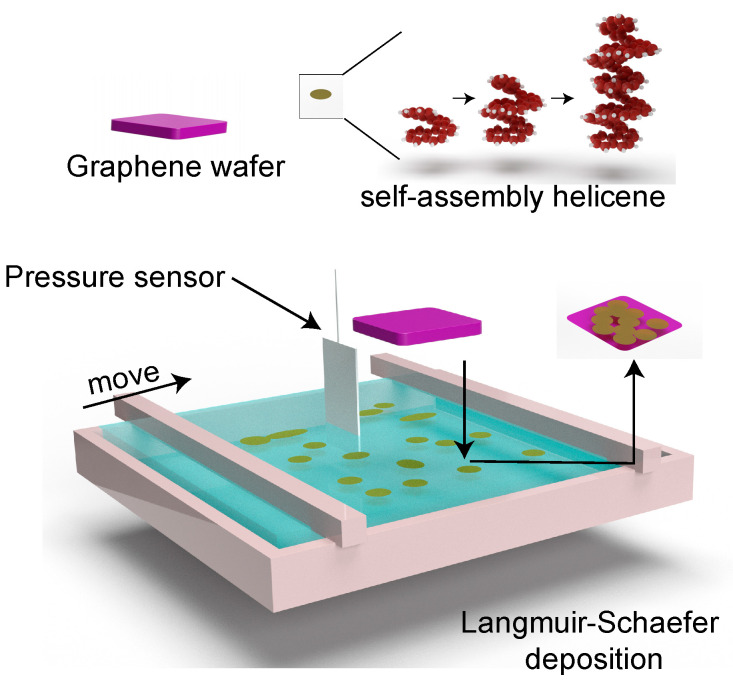
Illustration of the Langmuir-Schaefer deposition process of helicene on graphene. The surface pressure was measured using the Wilhelmy plate method with a disposable paper plate.

Helicene solution was prepared by dissolving 0.1% [7]helicene (purchased from Lach-Ner, Neratovice, Czech Republic, and used as received) in anhydrous dichloromethane via bath-sonication for 10 min at room temperature (23 °C). Then, 5 µL of helicene solution was spread at the air-water interfaces of a Langmuir trough (KSV Nima, Biolin Scientific, Gothenburg, Sweden) (Figure 1). The surface pressure was measured in the Langmuir trough via the standardized Wilhelmy plate method with a disposable paper plate. Upon evaporation of dichloromethane, a self-assembled helicene layer was deposited on the graphene monolayer (GR) that resides on a silicon wafer (Graphenea Inc., Cambridge, MA, USA) using the Langmuir-Schaefer method [38,39,40]. The deposited films were gently rinsed in dichloromethane to remove unadsorbed molecules.

The films were characterized by an atomic force microscope (AFM, Bruker Dimension Icon, Billerica, MA, USA) via the tapping mode at room temperature under atmospheric conditions. The measurements were performed with a silicon tip (OMCL-AC200TS-R3, Olympus, Center Valley, PA, USA), which has a radius of curvature of 7 nm, a spring constant of 9 N/m, and a resonant frequency of 150 kHz, at a scan rate of 0.5 Hz. The wetting behavior of water on the prepared films was determined under static conditions via the sessile drop technique. In these experiments, 5 µL of Milli-Q water was dropped on the surface of interest, then there was a 1 min wait for the dissipation of surface waves-induced by the impact of placement. The image of the water droplet was taken by a high-resolution camera (Moticam 1000, Motic, San Antonio, TX, USA) and analyzed through ImageJ software (National Institutes of Health (NIH), Bethesda, MD, USA) via Low-Bond Axisymmetric Drop Shape Analysis (LBADSA) with three replicate [27].

*S. aureus* (ATCC 13565) was prepared as described in previous studies [41,42]. Briefly, first, one microloop (10 µL) of bacteria was transferred to 9 mL tryptic soy broth (TSB) from a tryptic soy agar (TSA) slant. After incubating at 37 °C for 24 h, a second batch of *S. aureus* was prepared by transferring 10 µL of material from the first 9 mL of TSB with grown bacteria to a fresh 9 mL TSB followed by incubating at the same conditions. After 24 h of incubation, the working culture of *S. aureus* was purified by centrifuge at 1500× *g* for 15 min and resuspended in 0.1 wt% peptone water. This purification process was repeated three times. Finally, bacteria were suspended into 9 mL sterilized DI water to reach a final population of 8.8 ± 0.2 Log10 CFU/mL (6.3 ± 2.7 × 10^8^ CFU/mL) that was confirmed by the agar plating assay [15]. Then, helicene/graphene monolayer or bare graphene monolayer were inoculated with 2 mL of the *S. aureus* suspension for 4 h at room temperature. Afterward, the surfaces were removed from the suspension and rinsed by dipping and gently swirling in a sterile DI water reservoir three times to remove non-adhering bacterial cells and then air-dried for 30 min.

The areal density of *S. aureus* on the samples was determined using SEM (JSM-7500F, Jeol, Tokyo, Japan) and direct enumeration of bacteria from SEM micrographs. Before SEM imaging, the bacteria which dried after 30 min was followed by an inactivation process that was exposed to trace amounts of acrolein vapor. Afterward, a 5 nm layer of palladium and platinum (Pd/Pt) alloy was deposited on the surfaces to ensure the electrical conductivity for SEM measurement and immobilized adhered bacteria cells. Ten SEM images from random areas were collected from three samples and analyzed to quantify the attachment of *S. aureus.* To check the statistically different number of adhered bacterial on the sample with different deposit conditions, the counted bacterial density was statistically analyzed by one-way analysis of variance (ANOVA) with Tukey’s post-hoc test, using JMP software (SAS Institute, Inc., Cary, NC, USA) [27].

## 3. Results and Discussion

**Figure 2:**
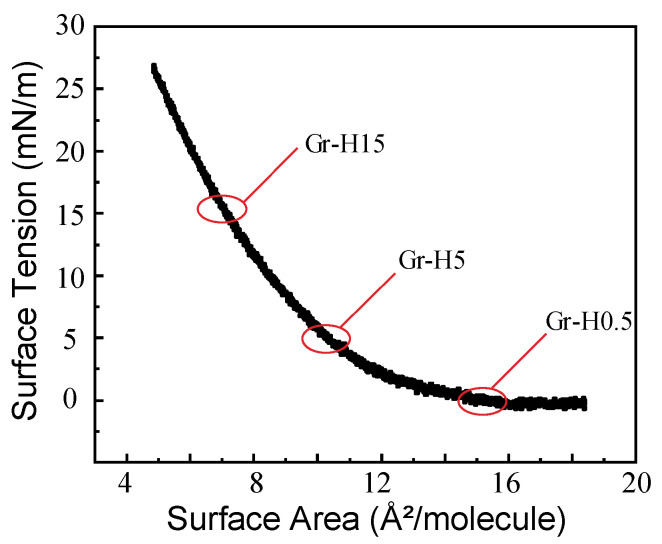
Pressure-area isotherm of [7]helicene at the air-water interface where three different surface pressures were used to prepare helicene films on graphene (GR-H15, GR-H5, and GR-H0.5).

Pressure-area isotherms of [7]helicene demonstrated a repulsion behavior with a gradually steeper slope as the degree of lateral confinement increases (Figure 2). Above a surface area per molecule of 16 Å^2^/molecule, the surface pressure was zero. When the spacing between molecules was decreased and the surface area per molecule reached ~10.5 Å^2^/molecule, the surface pressure increased to 5 mN/m. Surface pressure was tripled to 15 mN/m from 5 mN/m upon decreasing surface area per molecule from 10.5 to 7 Å^2^/molecule. The rapid increase in the surface pressure indicates a transition in the phase behavior of the interfacial film [43]. To investigate how the surface coverage influences the bacterial antifouling performance, samples with a different surface density of helicene was prepared at surface pressures of 0.5 mN/m (GR-H0.5), 5 mN/m (GR-H5), and 15 mN/m (GR-H15) on graphene. Bare graphene (GR) on a silicon wafer was used as a control surface.

**Figure 3:**
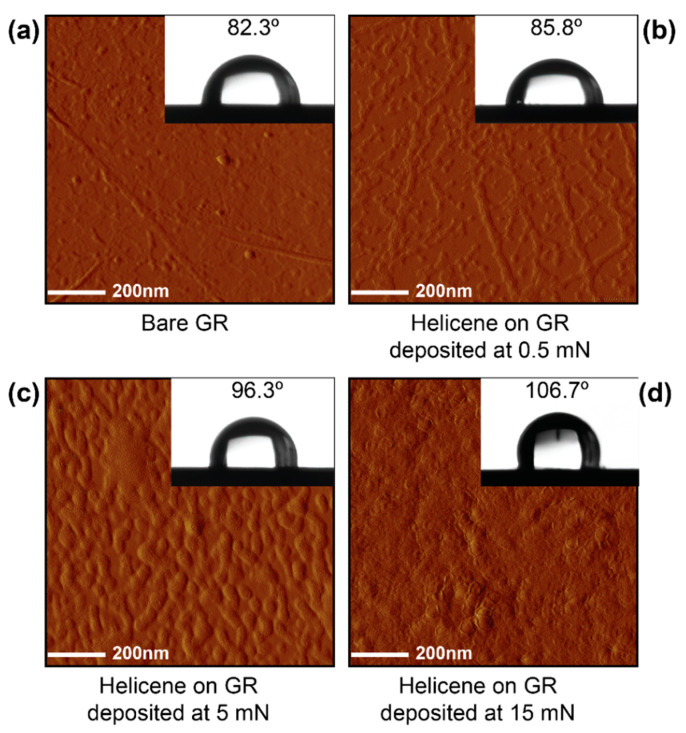
The amplitude error signal and static contact angle of water of/on (**a**) bare GR, (**b**) Gr-H0.5, (**c**) Gr-H5, and (**d**) Gr-H15. All images are 1 µm × 1 µm.

The amplitude error is an extremely sensitive mode of AFM for determining the shape of the topographical features of surfaces involving different phases. Figure 3a–d shows the amplitude error signal of bare GR and helicene coated GR at a surface pressure of 0.5 mN/m, 5 mN/m, and 15 mN/m. While the GR sample was relatively smooth as a whole, a few wrinkles could be observed. As the lateral compression pressure increased, the inter-domain distance of helicene self-assemblies decreased. At the surface pressure of 15 mN/m, the GR substrate was fully covered with helicene domains and more well-defined shape (hexagonal) domains were observed indicating an entropically-induced crystallization process. The amplitude error and the height sensor image can be found in Supporting Information Figure S1, which shows the uniformly covered surface with the condensed phase of the deposited helicene on the Gr-H15, Gr-H5, and Gr-H0.5 have a similar conclusion.

The static contact angle of water was 82.3° ± 1.3° on GR, indicating close to hydrophobic behavior, while it was 85.8° ± 2.5°, 96.3° ± 1.3°, and 106.7° ± 2.0° for Gr-H0.5, Gr-H5, and Gr-H15, respectively (Figure 3). Since the torsional geometry distorts the electron clouds of helicene [44,45], electrons in helicenes are less coupled/conjugated compared to graphene. The fully conjugated electron clouds interact favorably with water, which can be exemplified by the noticeable solubility of benzene in water and the insolubility of cyclohexane in water. Hence, helicenes are expected to be more hydrophobic than graphene-based films due to the torsional geometry distortion of the electron clouds of helicene. Meanwhile, the observed trends of increasing hydrophobicity from sample Gr-H0.5 to Gr-H15 can be explained by the increasing areal density of helicene on GR at higher surface pressures. Furthermore, the increase in the surface roughness can account for the increased contact angles [46].

**Figure 4:**
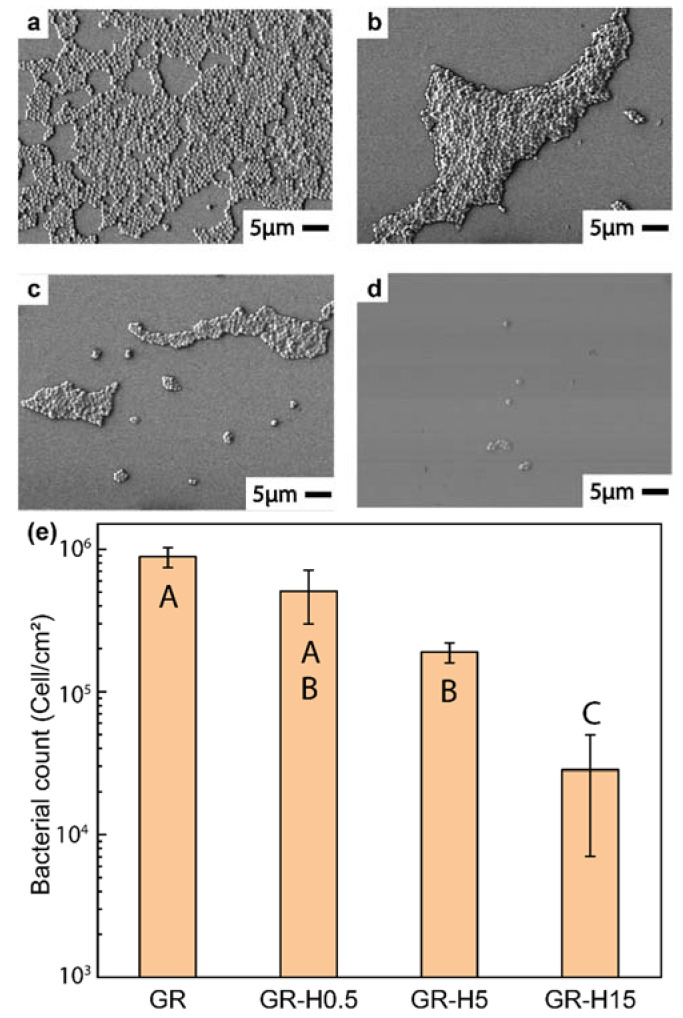
Scanning electron microscopy (SEM) micrographs of (**a**) GR (**b**) GR-H0.5, (**c**) GR-H5, and (**d**) GR-H15 samples after drop-cast inoculation with *S. aureus* for 4 h and (**e**) the corresponding bacterial counts obtained through the analysis of multiple micrographs. Statistically, the standard errors are reported based on ten random areas collected from three different samples. Letters A, B, C indicates statistically different *p*-value (*p* < 0.05).

The interactions of *S. aureus* with GR and helicene-deposited samples were investigated using SEM. As shown in Figure 4a–d, the bacterial adhesion significantly decreased as the coverage of helicene increased on the surfaces. Via a manual count from SEM micrographs, it was found that the number of adhering *S. aureus* on GR, GR-H0.5, GR-H5, and GR-H15 samples were 5.94 ± 0.07 log10 cell/cm^2^ (8.8 ± 1.4 × 10^5^ cell/mL), 5.67 ± 0.17 log10 cell/cm^2^ (5.0 ± 2.0 × 10^5^ cell/mL), 5.27 ± 0.07 log10 cell/cm^2^ (1.8 ± 0.3 × 10^5^ cell/mL), and 4.29 ± 0.46 log10 cell/cm^2^ (2.8 ± 2.1 × 10^4^ cell/mL), respectively. It is important to note that with SEM counting, it is not always possible to identify bacterial multilayers [47]. Hence, bacterial cell densities may be slightly higher if bacterial multilayers start to develop. Compared to the GR surface, the GR-H15 sample experienced a 96.8 ± 3.3% (31-fold) reduction in bacterial adhesion. In addition, a statistically significant difference between the population of adhered bacteria on GR-H15 and other conditions was confirmed with a *p*-value < 0.05 based on ANOVA with Tukey’s post-hoc test. 

Additional SEM micrographs can be found in Supporting Information, Figure S2.

Prior studies reported that the bacterial adhesion tends to increase as the substrate becomes more hydrophobic for hydrophilic bacteria [15,48,49]. In other words, for the adhesion of hydrophilic bacteria *S. aureus* to the hydrophobic surfaces (90° < θ < 150°), the number of adhering bacterial cells increases with an increasing contact angle [15,48,50,51]. This trend is valid until the substrate reaches the superhydrophobic region (θ > 150°) [27,52]. As such, the observed enhanced bacterial antifouling characteristics (on the more hydrophobic surface Gr-H15) cannot be simply ascribed to the surface hydrophobicity. On the other hand, it was reported that some surface textures can lead to the puncturing of bacteria, thereby decreasing bacterial fouling [36,53]. The significant reductions in bacterial adhesion on helicene coating may be ascribed to the molecular spring/screw structure of helicene [1], which can geometrically reduce the attractive van der Waals interactions between bacteria and substrate given that van der Waals forces are body forces [15,54]. In addition, the nanohelix structure can also introduce a steric barrier for bacterial docking.

## 4. Conclusions

The chiral compound with helical nanoarchitecture, [7]helicene, was deposited on graphene monolayers via the Langmuir-Schaefer method. The areal density of interfacially-assembled domains increased with increasing surface pressure, reaching a fully packed geometry at a surface pressure of 15 mN. Such helicene coated graphene films demonstrated notable ability to inhibit the bacterial adhesion, up to 96.8% (31-fold) reduction compared to bare graphene monolayers upon 4-h drop-cast inoculation with *S. aureus* suspension, which corresponds to a useful but moderate reduction. It is likely that the unique molecular geometry (i.e., nano-helix) of [7]helicene hinders the bacterial docking processes on the surface. Overall, this work reports a novel property of helicene-based materials: the ability of bacterial antifouling. This characteristic may be exploited in emerging coatings for biomedical devices.

## Supplementary Materials

The following are available online at https://www.mdpi.com/2079-4991/11/1/89/s1, Figure S1: The height image of (a) bare GR, (b) Gr-H0.5, (c) Gr-H5, and (d) Gr-H15. All images are 1 µm×1 µm. The height bar is 20 nm; Figure S2: The SEM image of S.aureus adhered to (a) bare GR, (b) Gr-H0.5, (c) Gr-H5, and (d) Gr-H15.
