# Supplementary material for: Bacterial Antifouling Characteristics of Helicene—Graphene Films
Source: Nanomaterials (Basel). 2021 Jan 3;11(1):89. doi: 10.3390/nano11010089 (PMC7830421; doi:10.3390/nano11010089)
Supplement: Supplementary file 1 [file nanomaterials-11-00089-s001.pdf]

Supplementary Materials: Bacterial Antifouling Characteristics of Helicene—Graphene Films

Shuhao Liu, Michael Bae, Li Hao, Jun Kyun Oh, Andrew R. White, Younjin Min, Luis Cisneros-Zevallos and Mustafa Akbulut

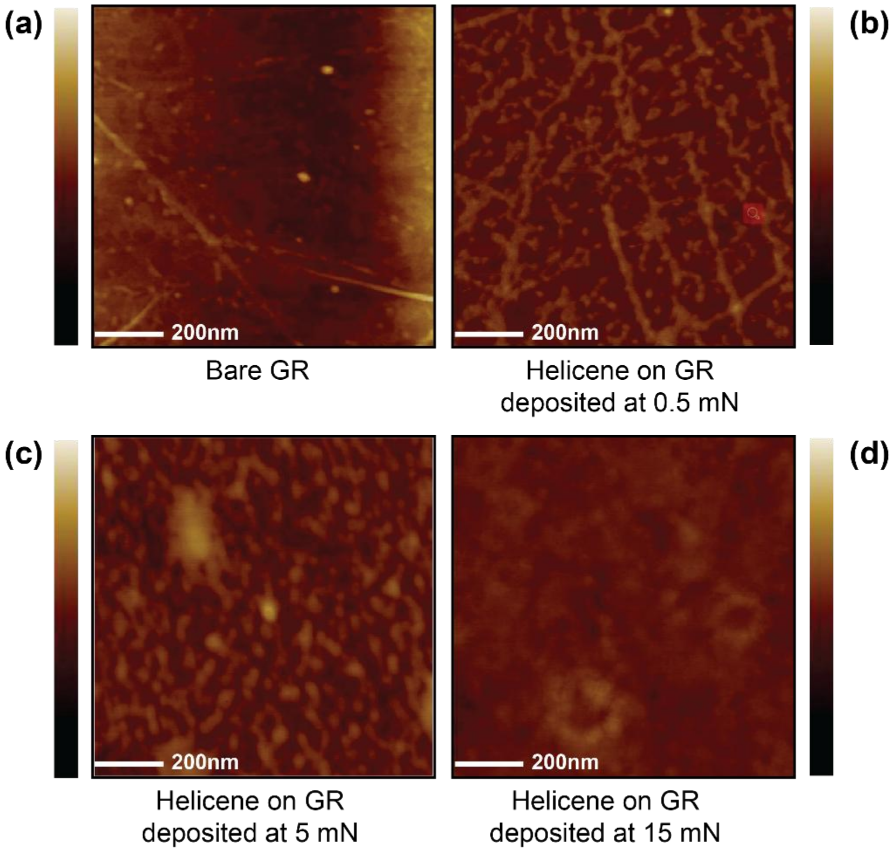

Figure S1. The height image of (**a**) bare GR, (**b**) Gr-H0.5, (**c**) Gr-H5, and (**d**) Gr-H15. All images are 1 μm ×1 μm. The height bar is 20 nm.

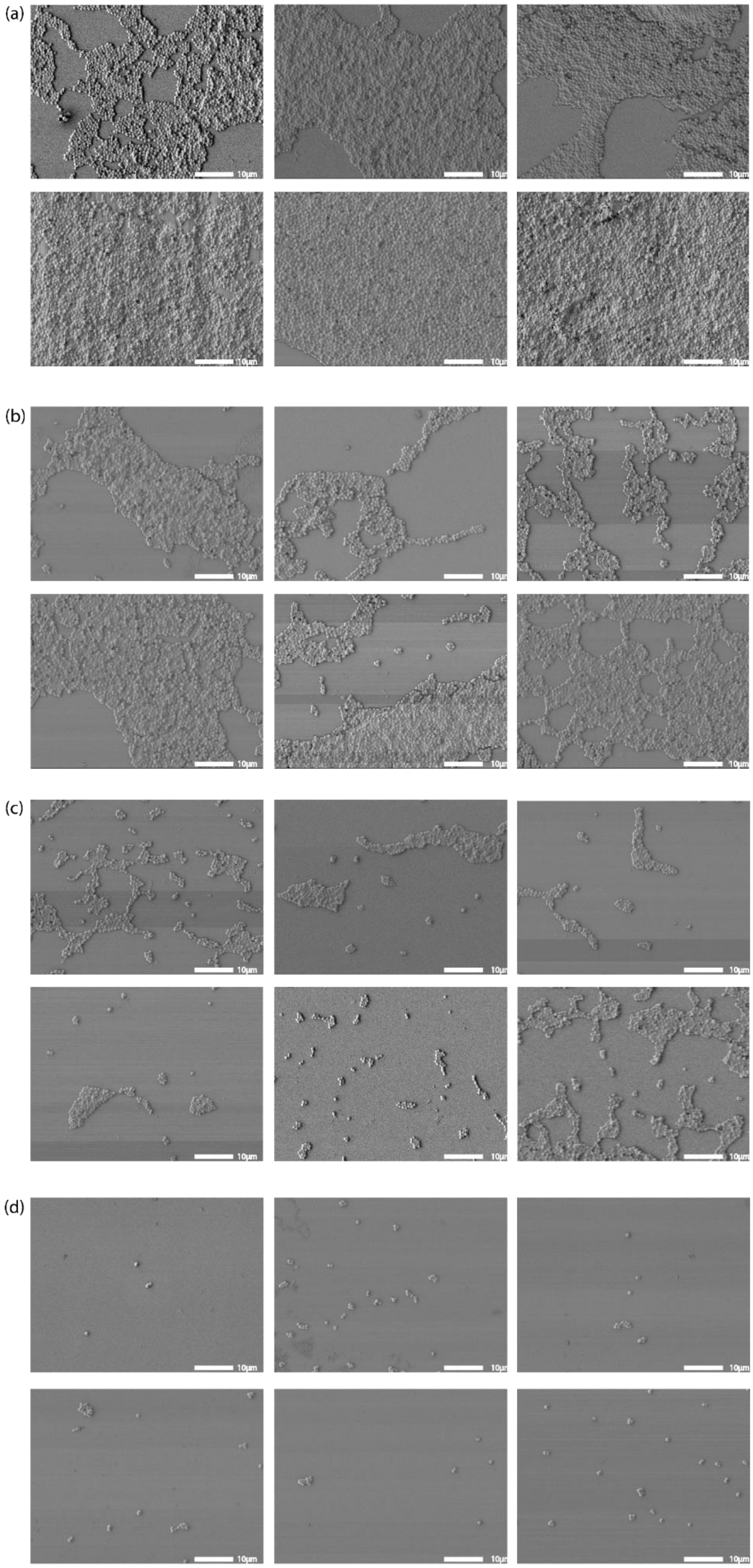

Figure S2. The SEM image of S.aureus adhered to (**a**) bare GR, (**b**) Gr-H0.5, (**c**) Gr-H5, and (**d**) Gr-H15.
